# Mid-life socioeconomic status, depressive symptomatology and general cognitive status among older adults: inter-relationships and temporal effects

**DOI:** 10.1186/s12877-016-0257-7

**Published:** 2016-04-20

**Authors:** Chi Chiao, Li-Jen Weng

**Affiliations:** Insitute of Health and Welfare Policy, Research Center for Health and Welfare Policy, College of Medicine, National Yang-Ming University, Taipei, Taiwan, R.O.C; Department of Psychology, College of Science, National Taiwan University, Taipei, Taiwan, R.O.C

**Keywords:** General cognitive status, Depressive symptomatology, Life course, Longitudinal studies, Socioeconomic factors, Taiwan

## Abstract

**Background:**

Few longitudinal studies have analyzed how socioeconomic status (SES) influences both depressive and cognitive development over an individual’s life course. This study investigates the change trajectories of both depressive symptomatology and general cognitive status, as well as their associations over time, focusing on the effects of mid-life SES.

**Methods:**

Data were obtained from the Taiwan Longitudinal Study on Aging (1993–2007), a nationally representative cohort study of older adults in Taiwan. The short form of the Center of Epidemiological Studies-Depression (CES-D) scale that measures depressive symptomatology in two domains (negative affect and lack of positive affect) was used. General cognitive status was assessed using the brief Short Portable Mental Status Questionnaire scale. Assessments of the subjects’ mid-life SES included measurement of the participant’s education and occupation. Analyses were conducted by the parallel latent growth curve modeling.

**Results:**

The participants’ initial levels of depressive symptomatology and general cognitive status were significantly and negatively correlated; furthermore, any changes in these two outcomes were also correlated over time. The initial assessment of general cognitive status significantly contributed to any advancement towards more severe depressive symptomatology over time, particularly when this occurred in a negative manner. Furthermore, a mid-life SES advantage resulted in a significant reduction in late-life depressive symptomatology and also produced a slower decline in general cognitive status during later life. In contrast, lower mid-life SES exacerbated depressive symptomatology during old age, both at the initial assessment and in terms of the change over time. In addition, female gender was significantly associated with lower general cognitive status and more severe depressive symptomatology in negative affect.

**Conclusions:**

These findings suggest a complex and longitudinal association between depressive symptomatology and general cognitive status in later life and this complicated relationship seems to be affected by mid-life SES over time.

## Background

Depression and cognitive impairment are among the most prevalent mental health problems for older people. Across studies using diverse samples and research methods, a cross-sectional association between poorer depressive symptomatology and poorer cognitive status among older adults has been well documented [1–6]. Research has also begun to explore the longitudinal relationship between the increase in depressive symptomatology and cognitive decline [3, 5–8] and these studies suggest that there are both temporal and progressive processes at work.

Despite the international mission to eliminate social disparities in mental health among the older population over the past few decades, longitudinal studies exploring depressive symptomatology and cognitive decline have not really addressed how socioeconomic status (SES) influences the development of depressive symptomatology and cognitive decline over an individual’s life course. SES is most commonly indexed via education and occupation, which is also widely used as a proxy for an individual’s cognitive reserve [9]. Existing findings, mainly using Western populations, have suggested that higher levels of education and occupation, namely a high SES, are protective against severe depressive symptomatology [10–12] as well as against a poor cognitive status [13, 14]. However, there has been some debate about how the impact of long-term SES differences is associated with the increase in depressive symptoms and the decline in cognitive status or how these associations vary over time [15–20].

The life-course hypothesis [21, 22] proposes that the accumulation of and/or interactions between various personal and SES environments/experiences throughout an individual’s life synergistically produce social disparities and health gradients during later years. Empirical work has linked a lower SES environment to an increase in depressive symptoms in later life [23–26], as well as with a decline in cognitive status [27–29]; furthermore, they have demonstrated that any disadvantages experienced during the adulthood of an individual would seem to exacerbate depressive symptomatology and produce a poorer cognitive status. Specifically, researchers have reasoned that an individual’s mid-life SES represents the accumulation of an individual’s exposure to socioeconomic stressors [30]. However, evidence from a life-course perspective is limited and this in turn has limited the exploration of the longitudinal impact of mid-life SES. It is important to consider simultaneously these impacts on any changes over time, namely both late life depressive increase and late life cognitive decline. This is especially true for non-western societies where such questions have been rarely addressed by researchers.

In this context, the stress process model [30] suggests that disadvantaged social groups are more likely than other groups to exhibit severe depressive symptomatology and a poor cognitive status because such groups disproportionately experience a range of adversities throughout their lives. A series of studies has also provided supporting evidence for the presence of gender disparities with respect to the symptoms of depression [10] and general cognitive status [13]. Among research conducted in non-western societies, women have been generally found to have more depressive symptoms than men [10]; in contrast, there have been mixed results regarding gender differences when general cognitive status has been examined [13, 27, 31].

As a result of knowledge gaps in previous studies, the present study aims to explore the longitudinal association between an increase in depressive symptomatology and a decline in general cognitive status among a cohort of older adults, as well as studying the influence of the individuals’ mid-life SES characteristics on the outcome trajectories of both of these two mental health measures. Based on prior studies [3, 5, 6], we first hypothesize that a poorer initial cognitive status of an individual contributes to the advancement of more severe depressive symptomatology over time in the same manner as the initial level of depressive symptomatology is associated with changes in general cognitive status over time. We further hypothesize that the progression of depressive symptomatology and any move towards a poorer cognitive status is affected by the individuals’ mid-life SES and that the aforementioned relationship may be partly contributed to by gender.

To be specific, we have examined whether a mid-life SES disadvantage at an initial assessment results in a lower general cognitive status and a higher level of depressive symptomatology compared to counterparts with a mid-life SES advantage at an initial assessment. In addition, we explored whether this SES disadvantage also results in a greater increase in depressive symptomatology and a greater decline in general cognitive status. Furthermore, do the aforementioned relationships remain even when there is adjustment for the influence of gender? We used a parallel latent trajectory model for this investigation, which is a powerful method that allows for a simultaneous modeling over time of the differences between and within individuals in both depressive and cognitive outcome changes.

## Methods

### The study population

The dataset for this analysis was obtained from the Taiwan Longitudinal Study on Aging (TLSA), a nationally representative sample. The baseline cohort was first interviewed in 1989 and included 4049 participants, 57 % men and 43 % women, who were aged between 60 and 96. However, measures of general cognitive status were not added to the survey until 1993. Therefore, this analysis has focused on data collected during 1993, 1996, 1999, 2003 and 2007. The analytical sample is restricted to adult respondents with complete self-reported data on two outcomes, general cognitive status and depressive symptomatology, yielding 2897 older adults in 1993, 2370 in 1996, 2032 in 1999, 1447 in 2003, and 978 in 2007. The study protocol used secondary data analysis of the TLSA and was approved by the Research Ethics Committee of National Yang-Ming University (Taipei, Taiwan).

Attrition is of special importance when carrying out longitudinal research in this population. As shown in our prior analysis [27], the group of participants who underwent attrition was more likely to be older and male, having poorer physical functioning, and smoke cigarettes. In addition, compared with those with a high SES, those who had missing data for their SES were more likely to be in the group that underwent attrition.

### Measurements

The two outcome measures in this study are depressive symptomatology and general cognitive status. Depressive symptomatology was measured via the 10-item short form of the Center of Epidemiological Studies-Depression scale (CES-D) [32]. Each item uses a 4-point scale to indicate how often each depressive symptom had occurred within the past week. The original 20-item CES-D has been widely used in survey research to assess emotional distress and has demonstrated satisfactory validity and reliability when used with Asian populations [24, 33]. Prior research and analyses reported herein has indicated that there are two distinct factor domains in this scale: negative affect and lack of a positive affect [24, 34, 35]. Negative affect included somatic and depressed affect items; lack of a positive affect consisted of positive items reversely coded. More detailed information on the psychometric properties of these two domains can be found in Chiao et al. [24]. Higher scores represent higher levels of depressive symptoms within each domain.

General cognitive status was measured by five items consistently across the TLSA waves and these items are part of a short portable mental status questionnaire (SPMSQ) [36]. The five items adopted were: ‘what are the day, the month, and the year’; ‘what day of the week is it’; ‘how old are you’; ‘what is your home address’; and ‘count backwards from 20 by 3 a total of four times’. For the last item, if the question was answered correctly all four times, then the task was recorded as having been completed correctly. The measure used for all the analyses was based on the count of correct answers [27, 37], resulting in a range of 0 to 5 positive answers. A higher score indicates a better general cognitive status (Cronbach’s α = 0.92–0.96). The use of these questions as cognitive tests has been validated for Chinese equivalents of the MMSE [38–40] and has also been used elsewhere as a measure of general cognitive status [37].

In addition to gender, another major explanatory variable is mid-life SES reported in later life and assessed by participant’s education and major adulthood occupation. Participant education was categorized into low (illiterate or incomplete primary education) or high (completed elementary school or higher) [28], and occupation was also categorized into low (manual, unemployed or housekeeper) or high (non-manual occupation) [41]. Individuals with a low level of education made up more than half (39 % illiterate, 16 % incomplete primary education) of the sample and, about seven-eighths of the sample (39 % manual, 47 % unemployed or housekeeper) was categorized as having a low level of occupation. Next, the education and occupation measures were added together to produce a composite index that had a range from 1 to 3. Participants with a high score for both measures, indicating a high SES, were coded as 3. Those who had a high score on only one factor were coded as 2 (medium SES), while those who have a low value for both measures were coded as 1, representing low SES.

### Statistical analysis

We employed parallel latent growth curve modeling to study the influence of mid-life SES characteristics on both outcome trajectories for depressive symptomatology and general cognitive status in later life. This modeling includes two repeated outcome measures with maximum likelihood estimation and was carried out using the structural equation modeling program in STATA 13. Prior analyses from the same cohort samples have suggested that late-life changes of both depressive symptomatology and general cognitive status have a linear form approximately [23, 27]. These two trajectories were therefore hypothesized as linear and assessed using the parallel process models [42, 43].

**Fig. 1:**
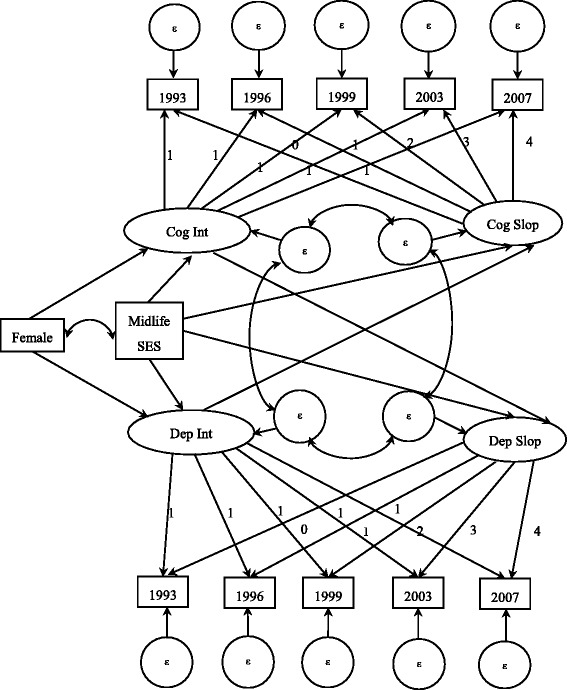
The parallel latent growth curve modeling of cognitive functioning and depressive symptomatology

As seen in Fig. 1, there are four latent factors identified as representing the initial assessments (intercepts) and the changes over time (slopes) of the trajectories of general cognitive status and depressive symptomatology within the two domains (negative effect and lack of positive effect). The latent intercepts with loadings fixed at 1 represent constant effects and the latent slopes, with loadings set from 0 to 4 across waves, represent a linear change over time. We modeled the aforementioned relationships separately for the two identified domains in depressive symptomatology based on prior research. The analyses reported herein support the existence of these two distinct factor domains of depressive symptomatology, namely a negative effect and a lack of positive effect [24, 27, 34].

Mid-life SES and gender are included as time-invariant covariates in order to investigate the inter-individual differences in SES and gender on the parallel growth trajectories. According to prior literature [26, 27], mid-life SES has been hypothesized to be an exogenous predictor both for initial assessments (intercepts) and for changes over time (slopes) when depressive symptomatology and general cognitive status are considered. Gender has been shown to directly involve with initial assessment. The correlation between mid-life SES and gender is also examined as part of this study.

## Results

### Descriptive findings

Table 1 provides sample descriptive statistics by wave. The results indicates that negative affect increased from 3.93 (SD = 4.96) in 1993 to 4.11 (SD = 4.93) in 2007; in contrast, level of lacking a positive affect decreased from 2.81 (SD = 2.49) to 1.79 (SD = 2.06) in 2007. Average scores for general cognitive status decreased from 4.34 (SD = 1.09) in 1993 to 3.91 (SD = 1.27) in 2007. Low mid-life SES individuals made up about three-fifths (58.1–61.7 %) of the sample, while about one-fourth of the sample (22.7–27.0 %) was categorized as high SES during mid-life across each wave.

**Table 1:** TLSA sample characteristics, 1993–2007

	1993 sample	1996 sample	1999 sample	2003 sample	2007 sample
	*N* = 2897	*N* = 2370	*N* = 2032	*N* = 1447	*N* = 978
	% or mean (SD)	% or mean (SD)	% or mean (SD)	% or mean (SD)	% or mean (SD)
Adult SES					
Low	61.65	61.10	59.94	58.12	57.67
Median	15.67	15.91	15.45	14.86	14.72
High	22.68	23.00	24.61	27.02	27.61
Gender					
Male	42.94	43.80	44.49	45.34	46.93
Female	57.06	56.20	55.51	54.66	53.07
Age	71.04 (5.65)	73.29 (5.10)	75.84 (4.85)	78.80 (4.06)	82.25 (3.73)
Cognitive functioning (0–5)	4.34 (1.09)	4.39 (1.03)	4.32 (1.08)	4.11 (1.16)	3.91 (1.27)
Psychological distress (CES-D)					
Negative Affect (0–24)	3.93 (4.96)	4.26 (5.24)	4.27 (5.34)	3.79 (4.94)	4.11 (4.93)
Lack of Positive Affect (0–6)	2.81 (2.49)	2.28 (2.42)	2.12 (2.24)	2.07 (2.24)	1.79 (2.06)

*SD* Standard deviation. Percentages may not add up to 100 due to rounding

### Relationship between cognitive decline and depressive symptomatology with respect to the negative affect domain

Table 2 presents the results from parallel latent growth curve modeling used to assess the effects of mid-life SES and gender on the longitudinal relationship between depressive symptomatology and cognitive decline. For the negative affect domain of depressive symptomatology, this model shows a good fit to the data (RMSEA = 0.031, CFI = 0.967, TLI = 0.956, and *χ*^2^ = 191.06, df = 55, *p* < 0.001). Although no significant association can be found between the depressive intercept and the cognitive status slope, the cognitive intercept significantly contributes to the depressive slope in the negative affect domain (β = 0.23, *p* < 0.001), indicating the effect of initial cognitive status on the change of negative affect over time. The intercepts of the cognitive status and the negative affect domain are negatively correlated (β = −0.76, *p* < 0.001); this means that older adults with a poorer cognitive status report a higher level of depressive symptoms in the negative affect domain at the initial assessment. The slopes of cognitive status and negative affect is also correlated (β = −0.06, *p* < 0.001).

**Table 2:** Results from the parallel growth curve model for cognitive functioning and depressive symptomatology

	Negative affect			Lack of positive affect		
	β		SE	β		SE
Direct effect						
Cognitive functioning						
Mid-life SES → Cognitive Intercept	0.37	^***^	0.03	0.37	^***^	0.03
Mid-life SES → Cognitive Slope	0.03	^*^	0.01	0.02		0.01
Female → Cognitive Intercept	−0.45	^***^	0.03	−0.46	^***^	0.03
Dep Intercept → Cognitive Slope	−0.001		0.004	−0.04		0.02
Intercept/slope covariance	−0.04	^**^	0.01	−0.04	^***^	0.01
Depressive symptomatology						
Mid-life SES → Depression Intercept	−0.76	^***^	0.13	−0.30	^***^	0.06
Mid-life SES → Depression Slope	−0.13	^*^	0.06	−0.05		0.03
Female → Depression Intercept	1.70	^***^	0.16	0.08		0.07
Cog Intercept → Depression Slope	0.23	^**^	0.08	0.04		0.04
Intercept/slope covariance	−0.31		0.25	−0.05		0.06
Covariance						
Covariance between intercepts	−0.76	^***^	0.08	−0.12	^***^	0.04
Covariance between slopes	−0.06	^***^	0.01	−0.01	^**^	0.01
Covariance between female and mid-life SES	−0.12	^***^	0.01	−0.12	^***^	0.01
Model Fitting						
*χ* ^2^ (55)	191.06	^***^		86.47	^***^	
RMSEA	0.031			0.016		
CFI	0.967			0.987		
TLI	0.956			0.982		
Latent factor	Mean		SD	Mean		SD
Depression Intercept	0.28		2.69	−0.14		0.51
Depression Slope	−0.07		0.32	−0.03		0.09
Cognitive Intercept	0.03		0.64	0.02		0.63
Cognitive Slope	0.02		0.09	0.01		0.08

^*^ *p* < 0.05, ^**^ *p* < 0.01, ^***^ *p* < 0.001. *SD* = Standard deviation

Mid-life SES has a direct effect on the intercepts of both general cognitive status (β = 0.37, *p* < 0.001) and the negative affect domain (β = −0.76, *p* < 0.001). Similarly, the mid-life SES has a direct effect on the slopes of both general cognitive status (β = 0.03, *p* < 0.05) and the negative affect domain (β = −0.13, *p* < 0.05). A SES advantage in mid-life contributes to a better general cognitive performance and reduces depressive symptomatology in the negative affect domain at initial assessments, as well as having the positive effect on their changes over time. In addition, there is a significant gender effect on the initial state of negative affect (β = 1.70, *p* < 0.001) and general cognitive status (β = −0.45, *p* < 0.001). Being a female is significantly associated with more severe depressive symptomatology in the negative affect domain and a poorer cognitive status.

### Relationship between cognitive decline and depressive symptomatology with respect to the lack of positive affect domain

Table 2 also presents the results for the lack of positive affect domain. The parallel latent growth curve model depicted in Fig. 1 also shows a good fit to the data (RMSEA = 0.016, CFI = 0.987, TLI = 0.982, and *χ*^2^ = 86.47, df = 55, *p* < 0.001). In contrast to the negative affect domain of depressive symptomatology, the cognitive status intercept does not significantly contribute to the depressive slope with the lack of positive affect domain and the depressive intercept is not associated with cognitive status slope, either. Mid-life SES has direct effects on the initial levels of cognitive status (β = 0.37, *p* < 0.001) and lacking positive affect (β = −0.30, *p* < 0.001). However, unlike the negative affect domain, mid-life SES does not have a significant effect on the slopes of cognitive status and the lack of positive affect. Furthermore, there is no gender difference in the initial level of the lack of positive affect domain.

## Discussion

The analyses carried out in this study have yielded a number of important findings. Firstly, the results of this study appear to support previous literature by showing that there is a cross-sectional relationship between depressive symptomatology and general cognitive status [4, 5, 8, 44] that an association between initial depressive symptomatology status and initial general cognitive status is found. Changes in depressive symptomatology and cognitive status over time have also been found to be related. Moreover, our results suggest a strong association between these two serious mental health conditions among older members of the community. A poorer initial general cognitive status seems to be related to a greater increase in the depressive symptomatology trajectory over time in the negative affect domain. The initial depressive symptomatology level, on the other hand, does not affect changes of cognitive status over time. Secondly, consistent with prior review-based research [10, 13], SES does matter. Our analyses have demonstrated a substantial effect of mid-life SES on the initial status of cognitive decline and depressive symptomatology for both domains, as well as a significant association between mid-life SES and changes in both general cognitive status and depressive symptomatology; this was true for the negative affect domain but not for the lack of positive affect domain. Finally, as also suggested by prior review-based research, the net effect of mid-life SES and gender was found to be associated with the initial assessments of depressive symptomatology for both domains [10, 45] and of general cognitive status [46]. Our results endorsed previous findings that being female and having a mid-life SES disadvantage is related to more severe depressive symptomatology and a poorer cognitive status in old age.

Some studies have shown that depressive symptomatology in a cohort is a precursor to cognitive decline [6, 8, 44, 47, 48]. However, our longitudinal analysis, that included five assessments of depressive symptomatology and general cognitive status using a cohort of older adult participants over 14 years, supports another set of studies showing an association between cognitive baseline status and depressive slope [3, 5, 7, 49]. In contrast, the depressive baseline in our study did not directly affect cognitive change, conflicting with prior reports [50]. However, that conclusion is limited to the fraction of baseline variance associated with depressive symptomatology, which is in turn related to mid-life SES and a female gender. The residual variance in the depressive baseline is indeed related to baseline cognitive status and through this possibly to the cognitive status slope.

Our findings substantiate the body of research that indicates an adverse effect of SES disadvantage on depressive symptomatology [51, 52] and cognitive status [28]. Such a SES gradient has been speculated to be associated with the presence of life-related stressors [30] that cause an increase in neuron loss via the glucocorticoid pathways [53]. In addition, our analyses also indicate that there is a significant effect of mid-life SES advantage with respect to changes over time in depressive symptomatology, particularly with respect to a negative affect; this is also true for cognitive decline with respect to mid-life SES disadvantage when the cohort becomes older. As suggested by the life-course hypothesis, individuals with a lower SES have limited socioeconomic resources and this in turn contributes to poorer long-term health [30]. Our findings supported the *accumulation effect* [21, 22], whereby the gaps in both cognitive decline and depressive symptomatology in terms of a negative affect clearly increase over time when individuals with a mid-life SES disadvantage and individuals with a mid-life SES advantage are compared. This deterioration continues among these socioeconomic disadvantaged individuals from mid-life on into their later years.

In addition, our findings suggest there are gender differences in symptoms of depression [10] and cognitive status [13, 46] among this Asian population. Compared to men of the same cohort, older women are more likely to suffer from higher levels of depression and poorer cognitive status. These social patterns have in part been attributed to differences in exposure to socially-based adversities such as gender-role socialization [54].

Our research has used parallel latent growth curve modeling to capture the development of two central mental health conditions over time in old age simultaneously. This analytical strategy also provides new information regarding the effects of gender and mid-life SES on depressive trajectory and cognitive trajectory in later life. Nevertheless, this work is not without limitations. Firstly, attrition is of great concern when carrying out longitudinal research because older adults are typically in poorer health than the younger population groups, making them the most difficult to retain over the complete study period. The exclusion of these individuals may have yielded a “healthier” analytical sample and introduced a “health effect”. Secondly, the individual controls used in the analyses are limited even though our findings are consistent with results across multiple studies. Thirdly, TLSA uses screening measurements for depressive symptomatology and general cognitive status. Our conclusions may be limited if they are to be applied to health promotion due to the use of screening instruments rather than clinical treatment. Fourthly, the SES measure used in the present analyses is a mid-life measure. Prior research has indicated such a variable is likely to have changed [51]. Any analysis of additional time-varying covariates was beyond the scope of this investigation, which has focused on the time-varying nature of SES and the relationship of these focal constructs with mental health. The next logical step in this line of inquiry is to investigate the role of changes in SES with respect to the pathway linking SES, depressive symptomatology, and cognitive decline.

## Conclusion

Despite the above limitations, to the best of our knowledge, our study is the first to report a temporal link between an initial poor cognitive status and a subsequent increase in depressive symptomatology over a 14-year follow-up period using an Asian population. To some extent, the trajectories of both depressive symptomatology and cognitive status over an individual’s life course are both further shaped by the individual’s mid-life SES and gender. This study shows that mid-life SES disadvantage and being female are precursors to more severe depressive symptomatology and poorer cognitive condition; furthermore, gender and mid-life SES also account for part of the temporal associations between the two mental health conditions. These findings are of crucial importance to world populations because the proportion of older individuals in many populations is increasing and the cost of treating mental health conditions in this expanding group may be rising. Policy-makers should take individual life-course SES and any related inequitable distribution of socioeconomic resources into consideration and begin developing programs and interventions that are aimed at promoting cognitive health and healthy aging among specific subpopulations.

### Availability of supporting data

The TLSA datasets supporting the conclusions of this article are made available under the approval from the Health Promotion Administration at Ministry of Health and Welfare in Taiwan in http://www.hpa.gov.tw/Bhpnet/Web/HealthTopic/Topic.aspx?id=200712250038.
